# Supplementary material for: Intracranial pressure and pulsatility in different head and body positions
Source: Brain Commun. 2025 Mar 19;7(2):fcaf115. doi: 10.1093/braincomms/fcaf115 (PMC11954551; doi:10.1093/braincomms/fcaf115)
Supplement: fcaf115_Supplementary_Data [file fcaf115_supplementary_data.docx]

**
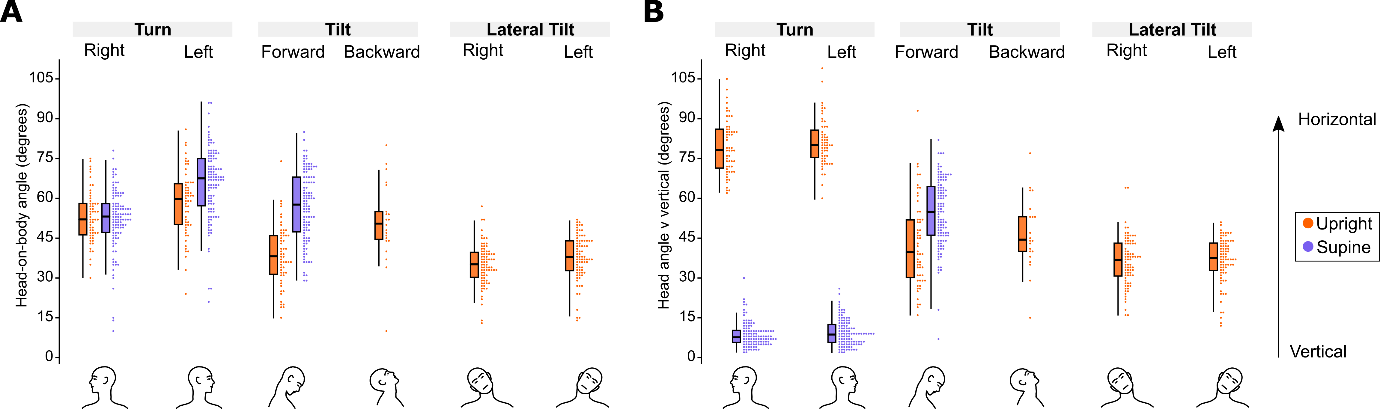
**

**Supplementary Figure 1. Head-on-body angle and head angle versus vertical in each head and body position.** A: Head-on-body angle in each head position for each body position. Head-on-body angle was computed as the change in head relative to body orientation from head neutral position (zero degrees; see Methods). B: Head angle relative to gravitational vertical. Note the head was approximately aligned with gravitational vertical (zero degrees) in head neutral-upright body positions but approximately 90 degrees relative to gravitational vertical (horizontal) in head neutral-supine body position. Head-on-body angle approximated the head angle versus vertical for head positions involving a predominant movement of the head in the vertical plane (tilt, lateral tilt). Purple=supine; orange=upright (seated, standing). Graphics below A and B aid interpretation of each head position. Filled circles are observations from individual participants; boxes represent the median (midline) and interquartile range (lower and upper bounds=25^th^, 75^th^ centiles, respectively); whiskers extend 150% of the interquartile range beyond the 25^th^ and 75^th^. N observations (n participants): upright[turn,forward tilt] N=108 (n=57); supine[turn,forward tilt] N=56 (n=56); upright[backward tilt] N=21 (n=21); upright [lateral tilt] N=78 (n=40)). Turn and forward tilt were measured in seated, standing and supine body positions. Lateral tilt was measured in seated and standing body position only. Backward tilt was measured in seated body position only.

**Supplementary Table 1.** **Change in head-on-body angle versus head neutral and head angle versus vertical in each head and body position.**

|  | Upright body position | | | | | | Supine body position | | |
| --- | --- | --- | --- | --- | --- | --- | --- | --- | --- |
|  | Turn | | Tilt | | Lateral Tilt | | Turn | | Tilt |
|  | Right | Left | Forward | Backward | Right | Left | Right | Left | Forward |
| n | 57 | 57 | 57 | 21 | 40 | 40 | 56 | 56 | 56 |
| Head-on-body angle (degrees) | 53  [47,59] | 69  [59,79] | 62  [49,71] | 50  [44,58] | 36  [29,39] | 41  [34,46] | 52  [46,58] | 60  [50,66] | 38  [31,46] |
| Head verticality  (degrees) | 7  [6,10] | 9  [6,13] | 57  [46,67] | 44  [39,54] | 37  [31,41] | 39  [34,43] | 78  [71,86] | 80  [75,86] | 40  [43,52] |
| Footnote: Values are whole cohort medians and interquartile ranges [25^th^,75^th^ centile]. Upright values represent the group statistics after mean averaging seated and standing data per participant. Turn and forward tilt were measured in seated, standing and supine body positions. Lateral tilt was measured in seated and standing body position only. Backward tilt was measured in seated body position only. Head verticality: 0=vertical; 90=horizontal. n=number of participants. | | | | | | | | | |

**Supplementary Table 2.** **Unadjusted change in ICP and pulsatility versus head neutral in each head and body position.**

|  | Upright body position | | | | | | Supine body position | | |
| --- | --- | --- | --- | --- | --- | --- | --- | --- | --- |
|  | Turn | | Tilt | | Lateral Tilt | | Turn | | Tilt |
|  | Right | Left | Forward | Backward | Right | Left | Right | Left | Forward |
| n | 57 | 57 | 57 | 21 | 40 | 40 | 56 | 56 | 56 |
| ICP  (mmHg) | 4.3±3.9  [3.2,5.3] | 3.6±4.3  [2.5,4.7] | 6.5±4.0  [5.4,7.5] | 7.4±6.9  [4.5,10.4] | 4.4±2.4  [3.6,5.1] | 2.8±2.5  [2.0,3.6] | 9.0±3.8  [8.0,10.0] | 5.7±3.4  [4.8,6.6] | 0.3±4.1  [-1.4,0.8] |
| Pulsatility  (mmHg) | 1.5±1.4  [1.1,1.9] | 1.4±1.6  [1.0,1.8] | -0.4±1.0  [-0.6,-0.1] | 2.8±4.4  [0.9,4.7] | 0.6±0.8  [0.3,0.8] | 0.5±0.7  [0.3,0.7] | 2.7±2.2  [2.1,3.2] | 2.1±2.2  [1.5,2.7] | 0.7±1.1  [0.43,1.0] |
| Footnote: Unadjusted head neutral (baseline) ICP: supine=12.1±5.7 mmHg [10.6,13.6], seated=2.2±6.7 mmHg [0.4,4.0], standing=2.3±6.1 mmHg [0.6,4.0]. Unadjusted head neutral (baseline) pulsatility: supine=4.2±2.0 mmHg [3.7,4.8], seated=5.2±3.0 mmHg [4.4,6.0], standing=4.6±2.2 mmHg [4.0,5.2]. Values are whole cohort means ± standard deviations and 95% CIs from raw data (not estimated or adjusted by statistical modelling). n=number of participants. Upright values represent group statistics after mean averaging seated and standing data per participant. Turn and forward tilt were measured in seated, standing and supine body positions. Lateral tilt was measured in seated and standing body position only. Backward tilt was measured in seated body position only. | | | | | | | | | |

**Supplementary Table 3. Effect of head turn and forward tilt on ICP in upright and supine body positions**

| Fixed effects: ∆icp | β | SE | z | P>z | [95% | CI] |
| --- | --- | --- | --- | --- | --- | --- |
| headonbodyangle | 0.039 | 0.012 | 3.135 | 0.002 | 0.015 | 0.063 |
| headposition |  |  |  |  |  |  |
| TurnLeft | -3.536 | 0.552 | -6.404 | 0.000 | -4.618 | -2.453 |
| TiltForward | -8.457 | 0.577 | -14.651 | 0.000 | -9.589 | -7.326 |
| bodyposition |  |  |  |  |  |  |
| Upright | -4.985 | 0.419 | -11.884 | 0.000 | -5.807 | -4.163 |
| headposition#bodyposition |  |  |  |  |  |  |
| TurnLeft#Upright | 2.158 | 0.567 | 3.808 | 0.000 | 1.047 | 3.268 |
| TiltForward#Upright | 10.170 | 0.609 | 16.710 | 0.000 | 8.977 | 11.363 |
| shunt#bodyposition |  |  |  |  |  |  |
| Function#Supine | -0.287 | 1.210 | -0.237 | 0.812 | -2.658 | 2.084 |
| Function#Upright | -0.678 | 1.151 | -0.589 | 0.556 | -2.935 | 1.578 |
| Malfunction#Supine | -0.026 | 1.104 | -0.024 | 0.981 | -2.190 | 2.137 |
| Malfunction#Upright | 3.258 | 1.039 | 3.136 | 0.002 | 1.222 | 5.294 |
| pots#headposition#bodyposition |  |  |  |  |  |  |
| 1#TurnRight#Supine | 3.585 | 2.233 | 1.605 | 0.108 | -0.792 | 7.962 |
| 1#TurnRight#Upright | 1.528 | 2.013 | 0.759 | 0.448 | -2.417 | 5.473 |
| 1#TurnLeft#Supine | 2.610 | 2.231 | 1.170 | 0.242 | -1.763 | 6.983 |
| 1#TurnLeft#Upright | 5.005 | 2.010 | 2.490 | 0.013 | 1.066 | 8.944 |
| 1#TiltForward#Supine | -2.692 | 2.233 | -1.205 | 0.228 | -7.069 | 1.685 |
| 1#TiltForward#Upright | 6.779 | 2.011 | 3.371 | 0.001 | 2.838 | 10.720 |
|  |  |  |  |  |  |  |
| Intercept | 6.776 | 0.874 | 7.754 | 0.000 | 5.063 | 8.489 |
| Random-effects parameters | Variance | SE |  |  | [95% | CI] |
| id | 5.943 | 1.411 |  |  | 3.732 | 9.465 |
| headposition | 2.618 | 0.608 |  |  | 1.661 | 4.126 |
| Residual | 5.367 | 0.423 |  |  | 4.600 | 6.263 |

Footnotes: Output of mixed effects model 1. Dependent variable: change in ICP relative to baseline (head neutral position) in head turn and forward tilt positions in seated, standing and supine body position. Fixed effects: head-on-body angle, head position, body position, head position x body position, shunt x body position, POTS x head position x body position; random effects: participant (id), head position. Number of observations: total=492; n=57 participants with mean 8.6 observations per participant (min=6, max=9) and mean 2.9 observations per participant per head position (min=2, max=3). Head-on-body angle in degrees. Shunt: functioning/malfunctioning/absent (base); head position: Turn Right (base)/Turn Left/Tilt Forward. Body position: supine (base)/upright. POTS: present (=1)/absent (=0; base). SE=standard error; CI=confidence interval. Model fit: AIC=2503; BIC=2587; R^2^=0.76; ICC [95%CI]: participant=0.42 [0.31,0.55], head position|participant=0.61 [0.52,0.70]. #=interaction.

**Supplementary Table 4. Effect of head turn and forward tilt on pulsatility in upright and supine body positions.**

| Fixed effects: Δpulsatility | β | SE | z | P>z | [95% | CI] |
| --- | --- | --- | --- | --- | --- | --- |
| ∆icp | 0.206 | 0.015 | 13.401 | 0.000 | 0.176 | 0.237 |
| headonbodyangle | -0.014 | 0.004 | -3.214 | 0.001 | -0.023 | -0.006 |
| headposition |  |  |  |  |  |  |
| TurnLeft | 0.301 | 0.245 | 1.228 | 0.219 | -0.179 | 0.782 |
| TiltForward | -0.377 | 0.279 | -1.351 | 0.177 | -0.924 | 0.170 |
| bodyposition |  |  |  |  |  |  |
| Upright | -0.252 | 0.179 | -1.411 | 0.158 | -0.602 | 0.098 |
| headposition#bodyposition |  |  |  |  |  |  |
| TurnLeft#Upright | 0.007 | 0.232 | 0.031 | 0.975 | -0.447 | 0.462 |
| TiltForward#Upright | -1.984 | 0.295 | -6.722 | 0.000 | -2.562 | -1.405 |
| shunt#headposition#bodyposition |  |  |  |  |  |  |
| Function#TurnRight#Supine | -1.194 | 0.508 | -2.351 | 0.019 | -2.190 | -0.199 |
| Function#TurnRight#Upright | -0.569 | 0.453 | -1.256 | 0.209 | -1.457 | 0.319 |
| Function#TurnLeft#Supine | -1.346 | 0.509 | -2.643 | 0.008 | -2.345 | -0.348 |
| Function#TurnLeft#Upright | -0.536 | 0.455 | -1.178 | 0.239 | -1.429 | 0.356 |
| Function#TiltForward#Supine | -0.239 | 0.508 | -0.471 | 0.637 | -1.234 | 0.756 |
| Function#TiltForward#Upright | 0.436 | 0.455 | 0.959 | 0.337 | -0.455 | 1.327 |
| Malfunction#TurnRight#Supine | 0.217 | 0.470 | 0.462 | 0.644 | -0.705 | 1.139 |
| Malfunction#TurnRight#Upright | 0.006 | 0.415 | 0.014 | 0.989 | -0.807 | 0.818 |
| Malfunction#TurnLeft#Supine | -0.068 | 0.470 | -0.144 | 0.885 | -0.989 | 0.853 |
| Malfunction#TurnLeft#Upright | -0.110 | 0.412 | -0.267 | 0.789 | -0.918 | 0.698 |
| Malfunction#TiltForward#Supine | 0.258 | 0.471 | 0.548 | 0.583 | -0.665 | 1.182 |
| Malfunction#TiltForward#Upright | -0.070 | 0.412 | -0.171 | 0.864 | -0.878 | 0.737 |
|  |  |  |  |  |  |  |
| Intercept | 1.674 | 0.323 | 5.191 | 0.000 | 1.042 | 2.306 |
| Random-effects parameters | Variance | SE |  |  | [95% | CI] |
| id | 0.378 | 0.122 |  |  | 0.201 | 0.713 |
| headposition | 0.472 | 0.096 |  |  | 0.317 | 0.703 |
| Residual | 0.686 | 0.054 |  |  | 0.588 | 0.801 |

Footnotes: Output of mixed effects model 2. Dependent variable: change in pulsatility relative to baseline (head neutral position) in head turn and forward tilt positions in seated, standing and supine body position. Fixed effects: change in ICP relative to baseline, head-on-body angle, head position, body position, head position x body position, shunt x head position x body position; random effects: participant (id), head position. Number of observations: total=492; n=57 participants with mean 8.6 observations per participant (min=6, max=9) and mean 2.9 observations per participant per head position (min=2, max=3). Head-on-body angle in degrees. Shunt: functioning/malfunctioning/absent (base); head position: Turn Right (base)/Turn Left/Tilt Forward. Body position: supine (base)/upright. SE=standard error; CI=confidence interval. Model fit: AIC=1497; BIC=1593; R^2^=0.79; ICC [95%CI]: participant=0.24 [0.14,0.40], head position|participant=0.55 [0.46,0.64]. #=interaction.

**Supplementary Table 5. Effect of head lateral tilt on ICP in upright body positions.**

| Fixed effects: Δicp | β | SE | z | P>z | [95% | CI] |
| --- | --- | --- | --- | --- | --- | --- |
| headposition |  |  |  |  |  |  |
| LatTiltLeft | -1.590 | 0.329 | -4.841 | 0.000 | -2.234 | -0.946 |
|  |  |  |  |  |  |  |
| Intercept | 4.281 | 0.392 | 10.920 | 0.000 | 3.512 | 5.049 |
| Random-effects parameters | Variance | SE |  |  | [95% | CI] |
| id | 3.979 | 1.162 |  |  | 2.244 | 7.053 |
| headposition | 0.689 | 0.527 |  |  | 0.154 | 3.082 |
| Residual | 2.853 | 0.460 |  |  | 2.080 | 3.913 |

Footnotes: Output of mixed effects model 3. Dependent variable: change in ICP relative to baseline (head neutral position) in head lateral tilt positions in seated and standing body position. Fixed effects: head position; random effects: participant (id), head position. Number of observations: total=156; n=40 participants with mean 3.9 observations per participant (min=2, max=4) and mean 1.9 observations per participant per head position (min=1, max=2). Head position: Lateral Tilt Right (base)/Lateral Tilt Left. SE=standard error; CI=confidence interval. Model fit: AIC=708; BIC=723; R^2^=0.65; ICC [95%CI]: participant=0.53 [0.36,0.69], head position|participant=0.62 [0.46,0.76].

**Supplementary Table 6. Effect of head lateral tilt on pulsatility in upright body positions.**

| Fixed effects: Δpulsatility | β | SE | z | P>z | [95% | CI] |
| --- | --- | --- | --- | --- | --- | --- |
| Δicp | 0.234 | 0.021 | 11.404 | 0.000 | 0.194 | 0.274 |
| headposition |  |  |  |  |  |  |
| LatTiltLeft | 0.347 | 0.085 | 4.090 | 0.000 | 0.181 | 0.513 |
|  |  |  |  |  |  |  |
| Intercept | -0.490 | 0.126 | -3.884 | 0.000 | -0.737 | -0.243 |
| Random-effects parameters | Variance | SE |  |  | [95% | CI] |
| id | 0.204 | 0.060 |  |  | 0.115 | 0.362 |
| headposition | 0.000 | 0.000 |  |  | 0.000 | 0.000 |
| Residual | 0.239 | 0.031 |  |  | 0.185 | 0.309 |

Footnotes: Output of mixed effects model 4. Dependent variable: change in pulsatility relative to baseline (head neutral position) in head lateral tilt positions in seated and standing body position. Fixed effects: change in ICP relative to baseline, head position; random effects: participant (id), head position. Number of observations: total=156; n=40 participants with mean 3.9 observations per participant (min=2, max=4) and mean 1.9 observations per participant per head position (min=1, max=2). Head position: Lateral Tilt Right (base)/Lateral Tilt Left. SE=standard error; CI=confidence interval. Model fit: AIC=290; BIC=308; R^2^=0.72; ICC [95%CI]: participant=0.46 [0.31,0.62], head position|participant=0.46 [0.31,0.62].

**Supplementary Table 7. Effect of head backward tilt on ICP in seated body position.**

| Fixed effects: Δicp10 | β | SE | z | P>z | [95% | CI] |
| --- | --- | --- | --- | --- | --- | --- |
| headposition |  |  |  |  |  |  |
| TiltBackward | -0.319 | 1.628 | -0.196 | 0.845 | -3.510 | 2.872 |
|  |  |  |  |  |  |  |
| Intercept | 7.725 | 1.231 | 6.276 | 0.000 | 5.313 | 10.138 |
| Random-effects parameters | Variance | SE |  |  | [95% | CI] |
| id | 3.992 | 6.998 |  |  | 0.129 | 123.996 |
| Residual | 27.827 | 8.588 |  |  | 15.198 | 50.951 |

Footnotes: Output of mixed effects model 5. Dependent variable: change in ICP relative to baseline (head neutral position) in head forward and backward tilt positions in seated body position. Fixed effects: head position; random effects: participant (id). Number of observations: total=42; n=21 participants with 2 observations per participant (one per head position). Head position: Tilt Forward (base)/ Tilt Backward. SE=standard error; CI=confidence interval. Head backward tilt position was measured over 10 s; the first 10 s of head forward tilt in seated body position was used for comparison. Model fit: AIC=272; BIC=279; R^2^=0.13; ICC [95%CI]: participant=0.13 [0.00,0.87].

**Supplementary Table 8. Effect of head backward tilt on pulsatility in seated body position.**

| Fixed effects: Δpulsatility10 | β | SE | z | P>z | [95% | CI] |
| --- | --- | --- | --- | --- | --- | --- |
| headposition |  |  |  |  |  |  |
| TiltBackward | 3.086 | 0.996 | 3.098 | 0.002 | 1.134 | 5.039 |
|  |  |  |  |  |  |  |
| Intercept | -0.353 | 0.704 | -0.501 | 0.617 | -1.733 | 1.028 |
| Random-effects parameters | Variance | SE |  |  | [95% | CI] |
| id | 0.000 | 0.000 |  |  | 0.000 | 0.000 |
| Residual | 10.421 | 2.274 |  |  | 6.795 | 15.983 |

Footnotes: Output of mixed effects model 6. Dependent variable: change in pulsatility relative to baseline (head neutral position) in head forward and backward tilt positions in seated body position. Fixed effects: head position; random effects: participant (id). Number of observations: total=42; n=21 participants with 2 observations per participant (one per head position). Head position: Tilt Forward (base)/ Tilt Backward. SE=standard error; CI=confidence interval. Head backward tilt position was measured over 10 s; the first 10 s of head forward tilt in seated body position was used for comparison. Model fit: AIC=225; BIC=232; R^2^=0.19; ICC [95%CI]: participant=0.00 [0.00,0.00].
